# Retraction Note: Multi-omics analysis of genomics, epigenomics and transcriptomics for molecular subtypes and core genes for lung adenocarcinoma

**DOI:** 10.1186/s12885-026-16489-y

**Published:** 2026-07-09

**Authors:** Yue Zhao, Yakun Gao, Xiaodong Xu, Jiwu Zhou, He Wang

**Affiliations:** 1 Department II of Radiotherapy, Cangzhou Central Hospital, No.16 Xinhua West Road, Cangzhou, Hebei 061110 China; 2 Department of Ultrasound, Cangzhou Central Hospital, Cangzhou, Hebei 061110 China; 3 School of Clinical Medicine, Cangzhou Medical College, Cangzhou, Hebei 061001 China; 4 Office of Educational Administration, Hebei Medical University, No.361 Zhongshan East Road, Shijiazhuang, Hebei 050017 China

**Retraction Note: BMC Cancer 21, 257 (2021)**

**https://doi.org/10.1186/s12885-021-07888-4**

The Editors have retracted this article. After publication, concerns were raised about some of the data in this Article. Specifically, similarities have been detected between.

• the lower part of the first panel (Control/0 h) and top part of the second panel (Empty vector/0 h) in the first row of Fig. 13A;

• the lower part of the second panel (Empty vector/0 h) and top part of the third panel (CNTN4/0 h) in the first row of Fig. 13A;

• the lower part of the first panel (Control/0 h) and top part of the second panel (Empty vector/0 h) in the first row of Fig. 13B; and.

• the lower-right part of the second panel (Empty Vector/0 h) and top-right part of the third panel (RFTN1/0 h) in the first row of Fig. 13B.

The Authors have not responded to these concerns. Therefore, the Editors have lost confidence in the data and conclusions of this Article.

Authors Yue Zhao, Yakun Gao, Xiaodong Xu, Jiwu Zhou, and He Wang did not respond to correspondence from the Publisher about this retraction.

